# In Vitro Immunoreactivity Evaluation of H-Ferritin-Based Nanodrugs

**DOI:** 10.1021/acs.bioconjchem.3c00038

**Published:** 2023-02-24

**Authors:** Leopoldo Sitia, Valentina Galbiati, Arianna Bonizzi, Marta Sevieri, Marta Truffi, Mattia Pinori, Emanuela Corsini, Marina Marinovich, Fabio Corsi, Serena Mazzucchelli

**Affiliations:** † Department of Biomedical and Clinical Sciences, Università degli studi di Milano, via G.B. Grassi 74, 20157 Milan, Italy; ‡ Department of Pharmacological and Biomolecular Sciences, Università degli studi di Milano, 20133 Milan, Italy; § Istituti Clinici Scientifici Maugeri IRCCS, 27100 Pavia, Italy

## Abstract

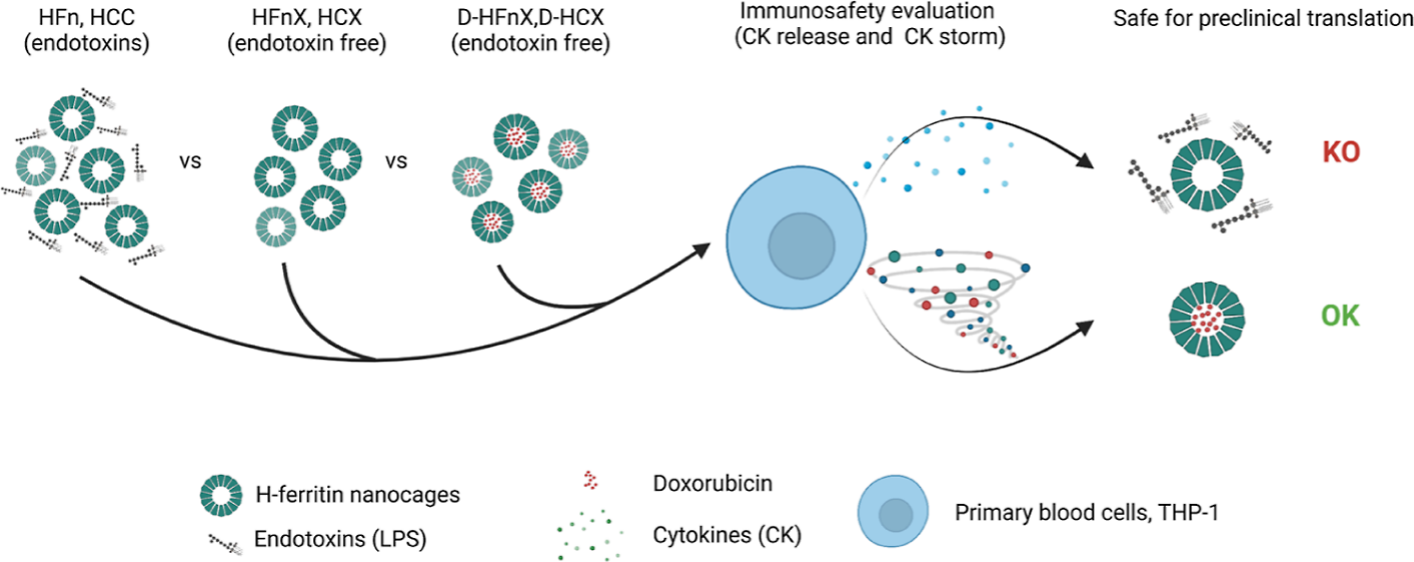

Biological nanoparticles, such as proteins and extracellular vesicles, are rapidly growing as nanobased drug-delivery agents due to their biocompatibility, high loading efficiency, and bioavailability. However, most of the candidates emerging preclinically hardly confirm their potential when entering clinical trials. Among other reasons, this is due to the low control of synthesis processes and the limited characterization of their potential immunoreactivity profiles. Here, we propose a combined method that allow us to fully characterize H-ferritin nanoparticles’ immunoreactivity during their production, purification, endotoxin removal, and drug loading. H-Ferritin is an extremely interesting nanocage that is being under evaluation for cancer therapy due to its innate cancer tropism, favorable size, and high stability. However, being a recombinant protein, its immunoreactivity should be carefully evaluated preclinically to enable further clinical translation. Surprisingly, this aspect is often underestimated by the scientific community. By measuring proinflammatory cytokine release as a function of endotoxin content, we found that even removing all pyrogenic contaminants from the nanocage, a mild immunoreactivity was still left. When we further purified H-ferritin by loading doxorubicin through a highly standardized loading method, proinflammatory cytokine release was eliminated. This confirmed the safety of H-ferritin nanocages to be used for drug delivery in cancer therapy. Our approach demonstrated that when evaluating the safety of nanodrugs, a combined analysis of acute toxicity and immunoreactivity is necessary to guarantee the safety of newly developed products and to unveil their real translational potential.

## Introduction

In the last 30 years, a lot of nanoparticles (NPs) and/or NP-based drugs have been developed.^1^ About 781,696 papers containing the word “nanoparticle” have been indexed in Scopus from 1970 to now. Despite this huge effort in research, only 31 of them have reached the clinics and the pharmaceutical market, while less than 100 are currently under investigation in clinical trials^2^ indicating a clear gap between research and clinics that should be filled. Therapeutic efficacy is the primary goal for nanotechnologists, and the quality and purity of NP samples are the issues often unconsidered.^3^ Surely, this issue is relevant when organic NPs are produced, while it is even crucial when we are dealing with protein-based NPs produced by fermentation in bacteria for in vivo experiments. Indeed, not many papers discuss the procedures of endotoxin quantification and removal when using organic and protein-based NPs because there are few works in which the production of NPs is followed by both in vitro and in vivo evidence.^4^ Endotoxins or lipopolysaccharides (LPSs) can influence the biological response of a treatment, for example, by stimulating the immune system since Toll-like receptor 4 (TLR4), which is the main LPS-signal transducer, is expressed not only by innate immune cells but also by several cell types, resulting in misinterpretation of biological results.^5^ Therefore, removing LPSs (and testing the final LPS content with a combination of precise assays) is of fundamental relevance to unveil the real efficacy of newly developed biological drugs or drug-delivery agents in vivo and before proceeding with further translation in human studies.^5^

To date, the evaluation of LPSs might not be sufficient to explain immunogenic reactions when using organic NPs as other nonpyrogenic contaminants can be found in solution. Moreover, NPs with different shapes and surface charges can be recognized as an exogen material that can stimulate cytokine release from monocytes leading to massive macrophage activation and phagocytosis.^6^

Here, we focused our attention on H-ferritin nanocages (HFn), a very promising protein-based class of NPs widely investigated as a targeted drug-delivery nanocarrier for cancer treatment.^7^ HFn is a 12 nm diameter shell that is able to enclose different molecules and anticancer agents, which displays natural tumor homing, thanks to the specific internalization mediated by the transferrin teceptor 1.^8^ HFn-based nanodrugs have been exploited for in vivo treatment of tumors, obtaining good results in terms of increased anticancer activity and reduction of off-target toxicity.^9−11^ Surpringly, among all research on HFn-based nanodrugs done by many different laboratories throughout the world, only a few publications have discussed the necessity of removing endotoxin contaminants from the protein.^12,13^ The rest of the experimental HFn-based nanodrugs are produced and tested without exploring any possible LPS contamination that might influence nanodrug response.

In this work, we reported our efforts to obtain LPS-free and pyrogen-free HFn from *Escherichia coli* fermentation, tuning both purification procedures (i.e., Triton X-114 removal of endotoxins) and the bacterial strain used for protein production, in order to finally obtain a nanodrug suitable for parenteral administration. Therefore, we compared the endotoxin content and immunoreactivity of HFn obtained by BL21(DE3) *E. coli* with or without Triton X-114 purification, with those obtained by the engineered ClearColi BL21(DE3) *E. coli* strain. Immunoreactivity was performed through the pyrogen test and cytokine release evaluation, and also doxorubicin (Doxo)-based nanodrugs were tested to confirm their suitability for drug-delivery purposes.

## Results and Discussion

### Production and LPS Evaluation of LPS-Free HFn (HFnX) in Comparison to Those of HFn

**Figure 1:**
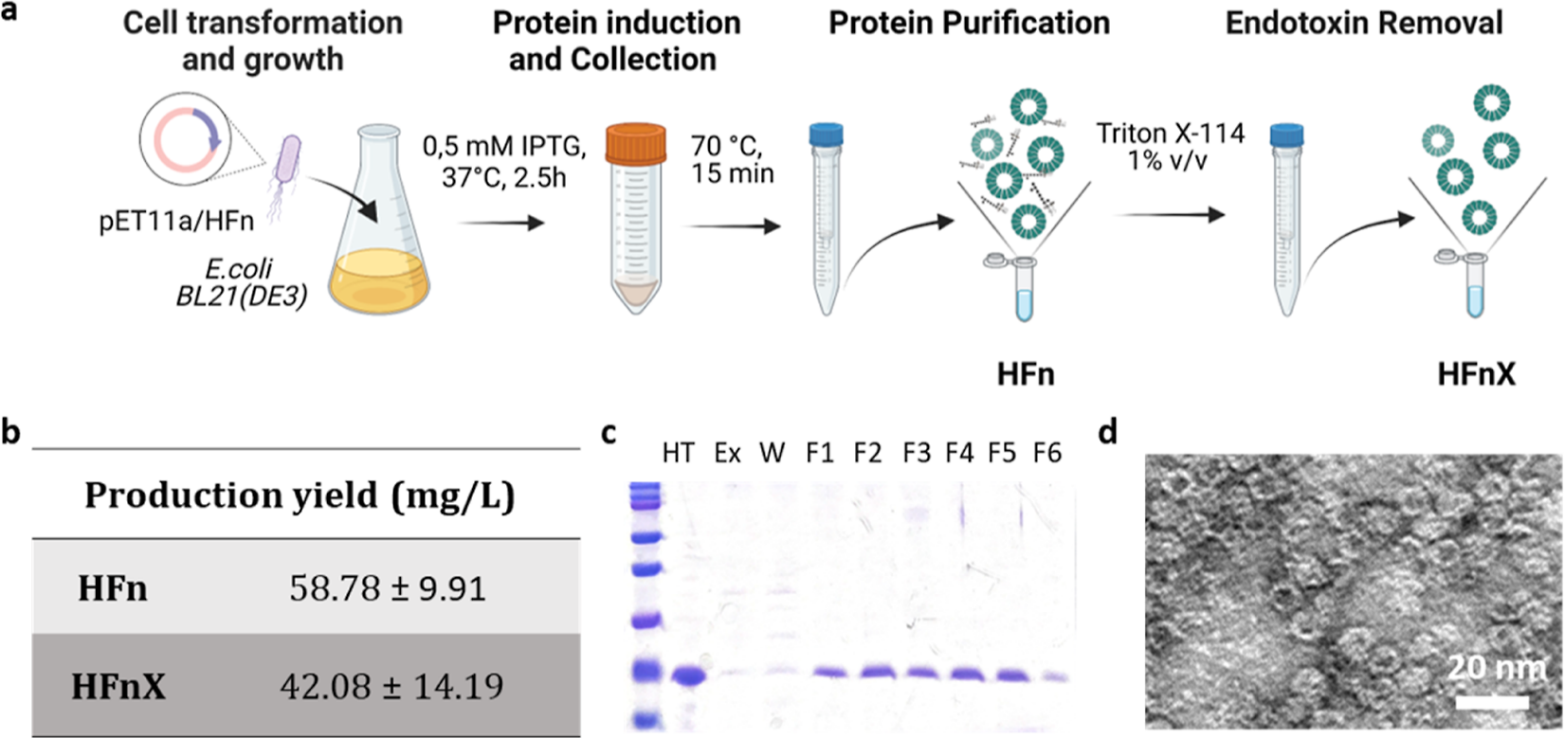
HFn and HFnX production methods (a); HFn and HFnX protein production yields (b); SDS-PAGE confirming the purity of HFnX monomers after production, purification, and LPS removal process (c); representative TEM image of HFnX confirming their spherical nanocage shape with an inner diameter of approximately 8 nm and an outer shell of 12 nm; scale bar 20 nm (d).

With the aim of preparing safe HFn-based nanodrugs, we first characterized the immunoreactivity of the bare protein used as a carrier. HFn was produced in BL21(DE3) *E. coli* strain and purified as already described in the literature and summarized in Figures 1a and S1(14) to obtain the final average protein yield of 58.78 ± 9.91 mg/L of culture (Figure 1b) with an average LPS content of 5.37 × 10^5^ ± 5.5 × 10^4^ endotoxin unit (EU)/mL corresponding to 1.94 × 10^5^ ± 1.91 × 10^4^ EU/mg (Table 1).

**Table 1:** Concentration of LPS before (HFn) and after (HFnX) Removal with Triton X-114

	LPS conc (EU/mg)	LPS conc (EU/mL)	LPS removal (%)
HFn	1.94 × 10^5^ ± 1.91 × 10^4^	5.37 × 10^5^ ± 5.5 × 10^4^	
HFnX	2.28 ± 2.04	8.34 ± 6.9	99.998

To remove LPSs from recombinant HFn, we applied the LPS removal protocol previously developed in our lab using Triton X-114, obtaining HFnX.^4^ Triton X-114 is water soluble at 4 °C and interacts with LPSs found in solution through electrostatic interactions. This process allowed the reduction of the LPS content down to 8.34 ± 6.9 EU/mL or 2.28 ± 2.04 EU/mg of HFnX (Table 1). This corresponded to 99.99% of LPS removal as compared to that of HFn. The final average HFnX production is 42.08 ± 14.19 mg/L (Figure 1b), with an average recovery of 71.59 ± 18.73%.

HFnX characterization by sodium dodecyl sulfate-polyacrylamide gel electrophoresis (SDS-PAGE) and transmission electron microscopy (TEM) confirmed that the Triton X-114 purification step did not affect the monomer size (Figure 1c) and that the process preserved the HFnX core–shell nanocage structure with an external diameter of approximately 12 nm (Figure 1d).

### Effect of HFn and HFnX on Cytokine Release in Human Primary Cells

**Figure 2:**
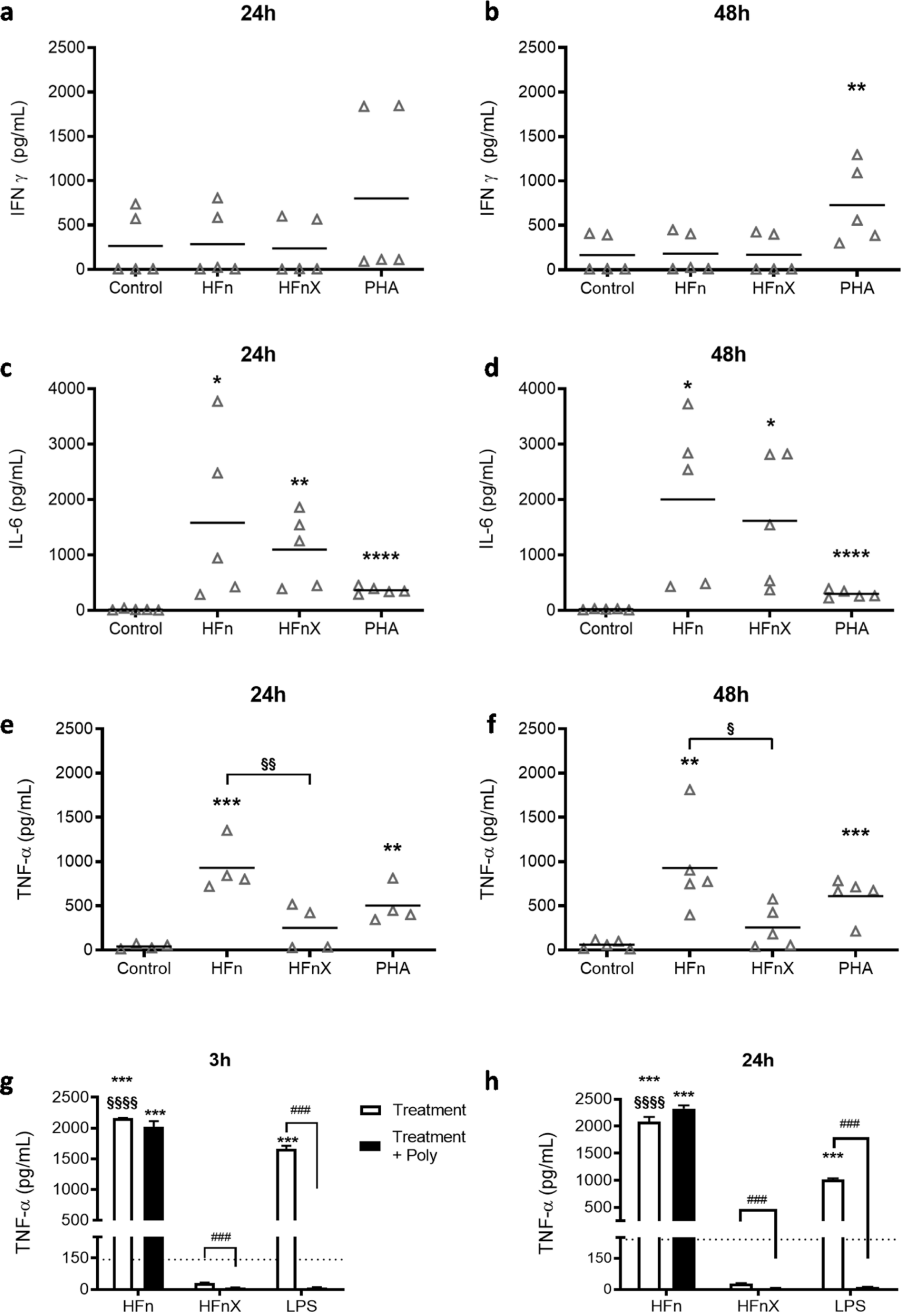
Effect of HFn and HFnX on cytokine release. Whole blood samples were diluted 1:10 in culture media and exposed to HFn (500 μg/mL), HFnX (500 μg/mL), and positive control PHA (5 μg/mL) for 24 and 48 h. IFN-γ (a,b), IL-6 (c,d), and TNF-α (e,f) release were assessed. Results are expressed as pg/mL. Each dot represents independent donors (*n* = 5). Statistical analysis was performed by one-way ANOVA, with *p < 0.05,**p < 0.01, and ****p < 0.0001 vs control; §*p* = 0.017 and §§*p* = 0.004 as indicated in the figure. Pyrogen test on THP-1 cells exposed to HFn (500 μg/mL), HFnX (500 μg/mL), and LPS (0.1 μg/mL) was performed, and TNF-α released was determined (white columns, pg/mL) after 3 and 24 h (g,h). Polymixin B pretreatment (black columns) was used to sequester LPS in solution and evaluate specific endotoxin-related immunoreactivity. Each column represents three independent experiments (*n* = 3). Statistical analysis was performed by one-way ANOVA, with ***p < 0.001 vs control, §§§§*p* < 0.0001 HFn vs HFnX groups, and Dunnett’s multiple comparison test, with ^###^*p* < 0.001 vs respective exposed groups.

In Figure 2, the immunoreactivity evaluation of HFn was performed using human primary and THP-1 cells. Whole blood samples obtained from five healthy donors were diluted 1:10 in culture media, and primary cells were treated with HFn and HFnX (500 μg/mL). Phytohemagglutinin (PHA—5 μg/mL) was used as a positive control.

No interferon gamma (IFN-γ) release was observed after HFn and HFnX exposure (Figure 2a,b). This result was particularly relevant for us as IFN-γ production, being related with macrophage activation, can promote NP phagocytosis, thus reducing HFn potential use as a drug-delivery agent.^15^ The release of proinflammatory interleukin 6 (IL-6) was significantly increased in both HFn and HFnX formulations even if the LPS removal procedure allowed a slight decrease of IL-6 levels but not comparable to the control group. Finally, tumour necrosis factor alpha (TNF-α) release was assessed. TNF-α levels measured by incubating HFn in human primary cells were significantly higher than those in untreated cells at both 24 and 48 h (Figure 2e,f), but in this case, the LPS removal performed in HFnX led to a significant decrease of TNF-α levels at both experimental time points as compared to HFn (Figure 2e,f).

Furthermore, a pyrogen test was performed using THP-1 cells and the two selected formulation previously mentioned, HFn and HFnX (500 μg/mL). LPS (0.1 μg/mL) was used as a positive control. Here, HFn was able to induce a statistically significant TNF-α release that was not modulated by polymixin B preincubation (Figure 2g,h). On the contrary, when incubating cells with HFnX, TNF-α levels were similar to untreated cells and significantly reduced as compared to HFn.

In summary, the results obtained with the whole blood assay and the pyrogen test indicate a proinflammatory role of HFn nanocages, supported by IL-6 and TNF-α release. After removing LPS with Triton X-114, this effect was reduced but not completely eliminated. The high cytokine activation observed after HFn incubation was somehow expected as it could be correlated with the high LPS contamination level. As already mentioned, this is generally not discussed in most papers that use HFn and other nanovectors as a delivery agent for anticancer drugs or imaging agents. Even if it is known that the immune response generated by LPS might have an influence on the observed activity profiles of the nanodrugs, authors tend to neglect this issue, and LPS removal processes are rarely presented.^5^ After Triton X-114 incubation, TNF-α release appears reduced, comparable with the control levels. However, a certain level of IL-6 activation was still observed. This could be explained with the fact that LPS concentration was reduced (less than 10 EU/mg) but not completely removed even by increasing the number of Triton X-114 cycles or by trying several commercially available affinity resins (data not shown). The persistency of the observed HFnX immunoreactivity, mainly represented by the high IL-6 release, led to a modification of the protein production and purification strategy, with the aim of using HFn as a safe drug-delivery agent.

### HCC Production in Clear Coli BL21 (DE3) Strain and Purification to Obtain HCX

**Figure 3:**
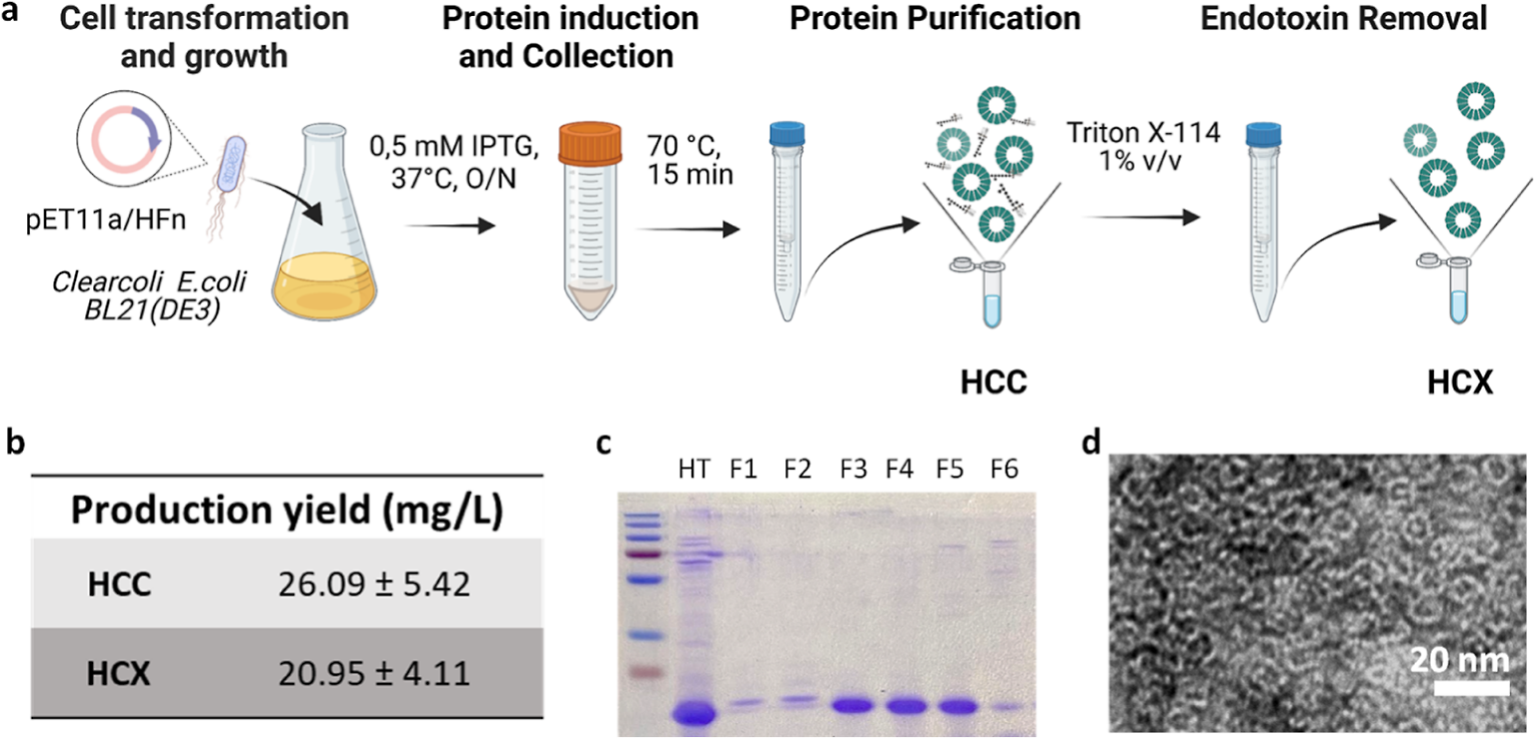
HCC and HCX production method (a); protein production yield for HCC and HCX (b). SDS-PAGE confirming the purity of HCX monomers after production, purification, and LPS removal process (HT: heat treated, F1-6: protein fractions) (c); representative TEM image of HCX confirms that the nanocage structure is comparable to that of other HFn-based nanocages (d).

To improve the immunoreactivity profile of HFn, we decided to abandon the production using BL21(DE3) and focused on the strain ClearColi BL21(DE3), an *E. coli* strain characterized by a genetically modified LPS that should significantly reduce its immunoreactivity.^16^ The method we used to produce and purify HFn in ClearColi (HCC) is summarized in Figure 3a and fully described in Figure S2. After trying different isopropyl β-*d*-1-thiogalactopyranoside (IPTG) concentrations and induction times, we selected the overnight (O/N) induction with 0.5 mM IPTG as it guaranteed the highest protein induction yield (Figure S3). As done for HFn, HCC was then purified by gel chromatography, followed by a final dialysis step, as already done for HFn. This process allowed us to obtain 26.09 ± 5.42 mg/L of protein (Figure 1b), reducing the yield of HCC by about 50% of that obtained for HFn. Gel electrophoresis confirmed the high purity and size of HCC monomers (Figure 3c), while TEM images highlighted that HCC maintained the HFn peculiar quaternary structure (Figure 3d).

Despite the use of an endotoxin-free engineered bacteria strain, limulus amebocyte lysate (LAL) test revealed the presence of 1.97 × 10^3^ EU/mg of LPSs (Table 2). To remove it, we decided to perform once again Triton X-114 purification to reach a LPS level below the limit imposed by the pharmacopoeia. After only two cycles of Triton X-114, we could obtain an LPS contamination of 0.87 ± 1.33 EU/mg. This was significantly less than that obtained with HFnX after four cycles of Triton X-114. After LPS removal, the average HCX production yield was 20.95 ± 4.11 g/L of cells (Figure 3d), corresponding to a final recovery of 81.13 ± 9.92%, slightly higher than the one obtained for HFnX. These results prompted us to test the immunoreactivity of HCC and HCX.

**Table 2:** Concentration of LPS before (HCC) and after (HCX) Removal with Triton X-114

	LPS conc (EU/mg)	LPS conc (EU/mL)	LPS removal (%)
HCC	1.97 × 10^3^ ± 2.4 × 10^2^	6.93 × 103 ± 7.6 × 102	-
HCX	0.87 ± 1.33	1.96 ± 2.96	99.96

### Effect of HCC and HCX on Cytokine Release in Human Primary Cells

**Figure 4:**
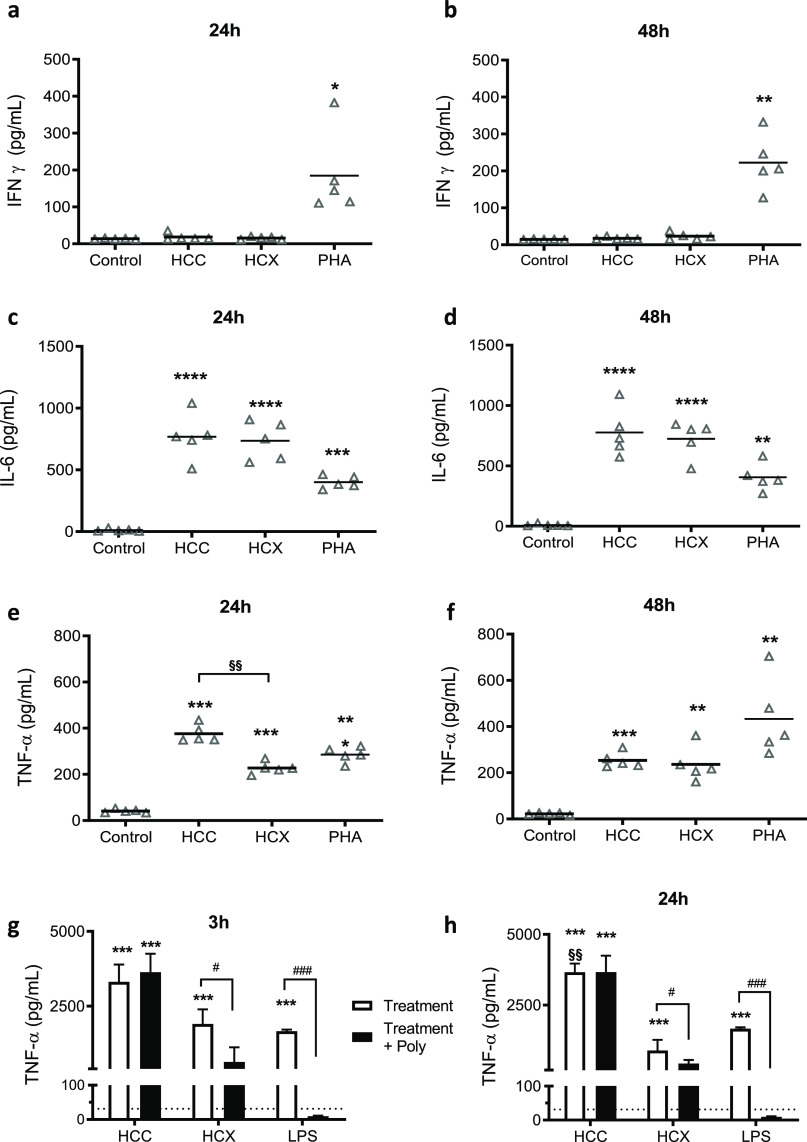
Effect of HCC and HCX on cytokine release. Whole blood was diluted 1:10 in culture medium and exposed to HCC (500 μg/mL), HCX (500 μg/mL), and positive control PHA (5 μg/mL) for 24 and 48 h. IFN-γ (a,b), IL-6 (c,d), and TNF-α (e,f) release were assessed. Results are expressed as pg/mL. Each dot represents independent donors (*n* = 5). Statistical analysis was performed by one-way ANOVA, with **p < 0.01, ***p < 0.001, and ****p < 0.0001 vs control; §§*p* = 0.0014 as indicated in the figure. Pyrogen test on THP-1 cells exposed to HCC (500 μg/mL), HCX (500 μg/mL), and LPS (0.1 μg/mL) was used to assess TNF-α release (white columns, pg/mL) after 3 and 24 h of incubation (g,h). Polymixin B pretreatment (black columns) was used to sequester LPS in solution and evaluate specific endotoxin-related immunoreactivity. Each column represents three independent experiments (*n* = 3). The dotted line represents control values (untreated cells). Statistical analysis was performed by one-way ANOVA, with ***p < 0.001 vs control, §§*p* = 0.001 HCC vs HCX, and Dunnett’s multiple comparison test, with ^#^*p* < 0.05 and ^###^*p* < 0.001 vs respective exposed group.

To investigate the effects of HCC and HCX in modulating cytokine release in human primary cells, the whole blood assay was repeated, as done for HFn and HFnX. After 24 and 48 h of exposure to HCC and HCX (500 μg/mL), no IFN-γ release was reported (Figure 4a,b). However, a statistically significant, not time-dependent, increase of IL-6 release was detected at both experimental time points (Figure 4c,d). IL-6 amount was around 50% of the levels obtained in HFn and HFnX, indicating a milder but persistent stimulatory effect despite endotoxin removal. TNF-α levels measured in HCC and HCX indicated a significant decrease in immunogenicity 24 h after incubation (Figure 4e). However, the cytokine levels were still significantly increased with respect to untreated cells at both 24 and 48 h (Figure 4e,f).

Similar results were found in the THP-1-based pyrogen test (Figure 4g,h), where both formulations induced a statistically significant increase in TNF-α release as compared to control levels. Interestingly, by removing the LPSs, we were only able to mildly reduce the cytokine release. Moreover, as polymyxin B was only partially able to silence TNF-α release, this suggested that the stimulatory effect obtained is only partially due to endotoxin contamination.

According to the literature, the LPS found in ClearColi strain is modified to make it pyrogen-free,^16^ and therefore, a certain level of immunoreactivity in HCC samples was expected. However, the cytokine release observed after removing modified LPS with Triton X-114 was unforeseen as LAL tests done on HCX ensured us that the level of endotoxin was below 1 EU/mg of protein. A reason that could explain the persistent immunoreactivity could be the high number of particles incubated with cells. In fact, the experiments were performed exposing cells to 500 μg/mL of HCX, corresponding to 6 × 10^14^ particles/mL. Even if the single particles are not immunogenic, literature evidence demonstrated that the high particle numbers are linked with cytokine activation.^17,18^ Taking into consideration that HCX will be used as a drug vector, our investigation continued focusing on the drug-loading process and loaded nanodrug’s immunoreactivity evaluation.

### Development and LAL Characterization of D-HFnX and D-HCX

**Figure 5:**
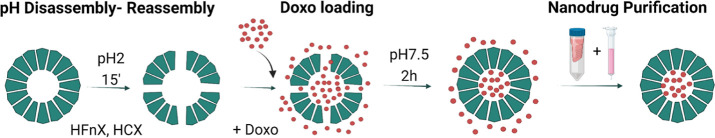
Protocol followed to load Doxo into HFnX and HCX. The nanocages are disassembled at pH 2 for 15′, then Doxo is added, and the nanocages are refolded at pH 7.5 and incubated for 2 h. At the end of incubation, the nanodrugs are concentrated, and the free drug is completely removed by gel filtration.

We have already used Doxo-loaded HFn nanocages as nanodrugs both in vitro and in vivo with very promising antitumor efficacy results.^9,10,14^ Here, for the first time, we are aimed at studying the immunostimulatory effect of the nanodrugs, testing the cytokine response after loading Doxo in both HFnX and HCX proteins (D-HFnX and D-HCX, respectively). The loading, described in Figure 5, was achieved using a pH disassembly-reassembly procedure, followed by incubation with the drug for 2 h and a final purification by size exclusion chromatography. As widely reported in the literature, HFn nanocages maintain their typical hollow spherical structure in a pH range between 3.5 and 10.^19,20^ This guarantees that the typical nanocage structure is maintained both within the slightly acidic peritumoral area (pH 6–6.5) and in the lysosomes (pH 5–5.5).^21−25^ To obtain a full nanocage disassembly, the reaction solution was brought at pH 2 and incubated with Doxo. After 15′, the pH was brought back to neutral, HFn and Doxo were incubated for 2 h, and the nanodrugs were further purified using proper molecular weight desalting columns.

As can be seen in Table 3, the average encapsulation rates were similar for both D-HFnX and D-HCX, namely, 14.1 and 15.4%, corresponding to an average of 33.42 and 33.79 molecules of Doxo encapsulated per nanocage. These values are in line with what was previously obtained by us and by other groups.^10,11^

**Table 3:** Detailed Characterization and Endotoxin Content of Nanodrugs

	% HFn rec.	% Doxo rec.	Doxo/HFn (mol no.)	EU/mg
D-HFnX	78.9 ± 19.8	14.1 ± 7.9	33.4 ± 13	4.5
D-HCX	87.2 ± 8.1	15.4 ± 8	33.8 ± 15.2	0.5

We tested the LPS content in the final products by the LAL assay, and we found that even if both nanodrugs had low levels of endotoxins, D-HCX was significantly less contaminated than D-HFnX (0.55 as compared to 4.5 EU/mg, Table 3). This was in line with the higher LPS residual content found in HFnX as compared to that in HCX.

### Cell Viability and Cytokine Storm Induced by Doxo, D-HFn, and D-HCX

Results obtained from the viability assessment (Figure S4) indicate a clear dose-response after Doxo, D-HFnX, and D-HCX exposure and a cytotoxicity effect starting from 10 μg/mL for the three substances tested, both at 24 and 48 h. As already demonstrated by our group, the toxicity observed was most likely due to the slow release of the loaded drug from the intact nanocages once they arrive inside the cells.^14^ On the contrary, the stability of HFn to a broad range of pH variation^26^ allowed us to exclude any issue related to Doxo release due to the disassembly of HFn nanocages.

**Figure 6:**
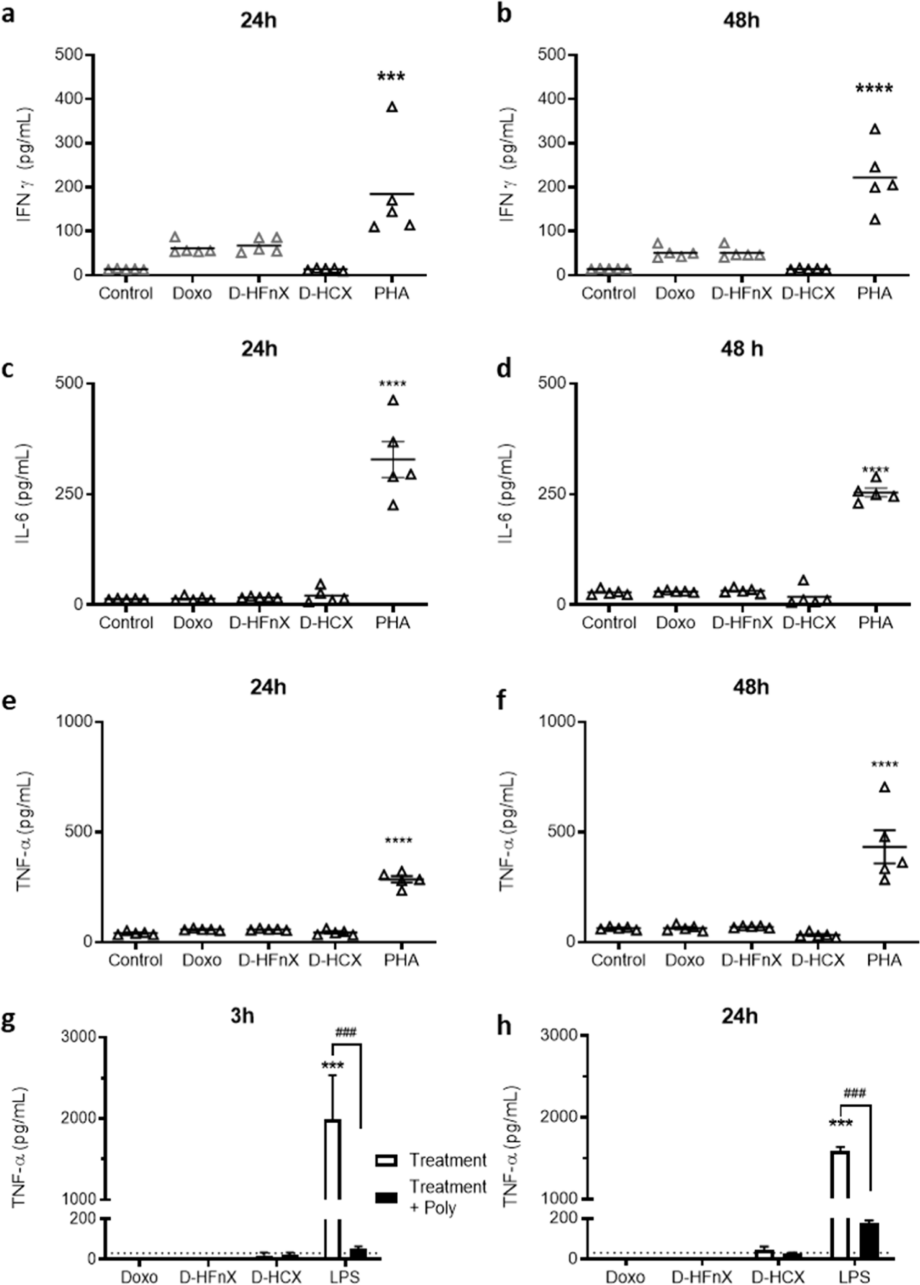
Effect of Doxo, D-HFnX, and D-HCX on cytokine release. Whole blood samples were diluted 1:10 in culture media and exposed to Doxo, D-HFnX, and D-HCX (3 μg/mL) for 24 and 48 h to assess cytokine release. PHA (5 μg/ml) was used as positive control. IFN-γ (a,b), IL-6 (c,d), and TNF-α (e,f) release were measured. Each dot represents independent donors (*n* = 5). Statistical analysis was performed by one-way ANOVA, with ***p < 0.001 and ****p < 0.0001 vs control. Pyrogen test on THP-1 cells exposed to Doxo, D-HFnX, and D-HCX (3 μg/mL) and LPS (0.1 μg/mL) was used to assess TNF-α release (white columns, pg/mL) after 3 and 24 h of incubation (g,h). Polymixin B pretreatment (black columns) was used to sequester LPS in solution and evaluate specific endotoxin-related immunoreactivity. Each column represents three independent experiments (*n* = 3). The dotted line represents control values (untreated cells). Statistical analysis was performed by one-way ANOVA, with ***p < 0.001 vs control, and Dunnett’s multiple comparison test, with ^###^*p* < 0.001 vs respective exposed group.

Starting from this evidence, the cytokine storm assessment was conducted using only the lowest concentration (3 μg/mL) for all substances (Figure 6). What emerged from the results is an overall decrease in the release profiles of all tested cytokines. All values were comparable with untreated cells at both time points, and no statistically significant differences were observed (Figure 6a–f).

Also the pyrogen test supports these results (Figure 6g,h), and considering the shorter incubation time, compared to the cytokine storm assay, the assay was conducted on all nanodrug concentrations used for cytotoxicity evaluation (Figure S5). No statistically significant difference was reported when incubating THP-1 cells with either 10 or 30 μg/mL of any of the testing agent (Doxo, D-HFnX, and D-HCX).

These results were quite surprising, given the immunoreactivity induced by HFnX and HCX empty nanocages. The loading protocol involves three different steps that might have contributed in modulating the cytokine release: (I) first protein was diluted down to 0.5 mg/mL in certified nonpyrogenic saline solution; (II) a further concentration and (III) a final buffer exchange using gel chromatography were performed. All these processes have contributed in further cleaning the protein from apparently nonpyrogenic contaminants (LPS levels did not vary before and after drug loading) that were however important in determining a reaction in both primary cells and the THP-1 cell line. Further studies should be anyway performed to fully characterize the different immunoreactivity of our nanocomposites at different stages of development.

## Conclusions

The present work was aimed at evaluating the immunoreactive profile of a new class of HFn-based nanodrugs to be used for drug delivery in cancer therapy.

Our results demonstrate that when working with nanodrugs, both the initial choice of the material and all production and purification steps should be carefully optimized to guarantee a final product that can be safely used for preclinical experiments and eventually translated for clinical studies.

In particular, we showed the following:

- Removal of LPS under the threshold defined by pharmacopoeia is necessary but not sufficient to avoid cytokine release and pyrogenic reactions.
- An assay strategy that combines LAL assay, evaluation of cytokine release, and pyrogenic test has been defined.
- This assay strategy should be fulfilled before planning in vivo studies, especially with protein-based and organic NPs.
- Further studies should be performed to better understand the immunoreactivity observed with LPS-free HCX nanocages before and after loading with Doxo.

## Experimental Procedures

### HFn and HFnX Production

HFn has been produced as a recombinant protein following a previously optimized protocol.^14^ Briefly, the pET11a/HFn plasmid was subcloned into BL21(DE3) *E. coli* that were grown until an OD_600nm_ = 0.6 in LB-Miller broth supplemented with ampicillin at 100 μg/mL. Gene expression was then induced with 0.5 mM IPTG (cat no. I1284, Sigma-Aldrich). At the end of incubation, the cells were centrifuged, collected, and lysed by sonication and heat shock. The extracted protein was then purified by ion-exchange chromatography using a DEAE Sepharose resin (cat no. DCL6B100, Sigma-Aldrich) and dialyzed overnight in PBS at 4 °C.

To remove endotoxins from purified HFn, we followed a protocol we recently set up, with slight modifications.^4^ Briefly, Triton X-114 was added to the HFn solution at a 1% v/v concentration in 15 mL tubes. The suspension was left at 4 °C on a tube rotator gently rotating for 30 min, incubated on a water bath at 37 °C for 15 min, and centrifuged at 37 °C for 15 min at 4900*g*. At the end of this process, two phases were formed inside the tubes. Triton X-114 and LPS were precipitated at the bottom, while HFn remained in the supernatant. HFn was carefully collected in a new tube, and the process was repeated three more times to further increase the LPS removal efficiency.

HFn and LPS-free HFn (HFnX) purity was assessed by SDS-PAGE (12% gel with a Coomassie brilliant blue protein stainer), and protein concentration was measured by absorbance reading (A280 nm). HFn physicochemical properties were evaluated by TEM.

The LPS content in the protein formulations was evaluated using the LAL kinetic turbidimetric assay following manufacturer’s instructions (Charles River Microbial Solutions Ltd., Dublin, Ireland).

### HCC and HCX Production

ClearColi BL21 (DE3) strain was purchased from Lucigen (LGC Ltd. UK). ClearColi BL21 (DE3)/pET11a/HFn transformed cells were plated on LB-Miller agar supplemented with ampicillin at 100 μg/mL (cat no. A0166, Sigma-Aldrich) and incubated O/N. A single colony of cells was collected and used to induce the growth of the preinoculum in LB-Miller supplemented with ampicillin at 100 μg/mL. The preinoculum was grown at 37 °C with shaking at 100 rpm overnight. The next day, the OD_600nm_ value reached by the preinoculum was determined. An adequate volume of cells was inoculated in 1 L of LB-Miller medium supplemented with ampicillin at 100 μg/mL to obtain an initial OD_600nm_ of 0.05. The incubation proceeded at 37 °C with constant stirring until reaching an OD_600nm_ of 0.6. After trying different conditions, gene expression was induced by the addition of 0.5 Mm IPTG, and the cells were further grown under constant stirring (100 rpm) at 37° C O/N. The cells were harvested by centrifugation at 4000*g* for 15 min at 4 °C. The pellet was then resuspended in physiological buffer, pH 7.2 (10 mM K_2_HPO_4_, 1.8 mM KH_2_PO_4_, 150 mM NaCl), recentrifuged at the same condition, and stored at −20 °C.

Before proceeding with ClearColi HFn (HCC) purification, the cells were thawed and resuspended in a lysis buffer (20 mM KMES pH 6.0, 1 mM phenylmethanesulfonyl fluoride, complete EDTA-free protease inhibitors (50×), 1 mg/mL lysozyme, and 20 mM MgCl_2_; 3 mL/g of cells). Then, DNAse (40 U/g of cells, cat no. DN25, Sigma-Aldrich) was also added, and the mixture was incubated for 30 min at 4 °C (on ice, shaking occasionally or on a wheel in a cold room). After that, the cells were disrupted by sonication (6 cycles of 10 s on ice). The cell lysate was centrifuged at 10,000*g* for 30 min at 4 °C, and the supernatant was collected and subsequently heat-treated at 70 °C for 15 min and then centrifuged at 10,000*g* for 30 min at 4 °C. The recovered supernatant was purified by ion-exchange chromatography using DEAE Sepharose resin (cat no. DCL6B100, Sigma-Aldrich, bed volume 5 mL). Elution was carried out with an increasing step-wise gradient of NaCl. The six fractions obtained were dialyzed overnight in PBS at 4 °C using dialysis cassettes (SLIDE-A-LYZER 20KD 12 mL, Fisher Scientific), and the protein content was dosed.

LPS were efficiently removed by incubating HCC with two cycles of Triton X-114, as described above. LPS quantification and protein characterization in HCC and HCX were performed using the same methods described for HFn and HFnX.

### HFnX and HCX Loading with Doxo

To prepare D-HFnX and D-HCX, Doxo was loaded using the pH disassembly-reassembly method already described by our group, with slight modifications.^14^ The protein was diluted down to 0.5 mg/mL into a 150 mM NaCl solution, adjusted to pH 2 to disassemble protein nanocages, and incubated at 180 rpm at room temperature (RT). After 15′, Doxo (200 μM) was added, the pH was adjusted back to 7.5, and the mixture was incubated for 2 h in agitation (180 rpm, RT). At the end of incubation, the solution was centrifuged (3500*g*, 15′) through 4 mL of 100 kDa Amicon membranes (Millipore) several times to simultaneously concentrate the nanodrug and remove nonencapsulated Doxo.

Finally, the nanodrugs were centrifuged through 7K MWCO Zeba Spin Desalting columns (Thermo Fisher) previously equilibrated with PBS for buffer exchange and further purification. Encapsulated Doxo was extracted by diluting the samples in a 1:1 isopropanol/chloroform solution, with SDS 0.01% and K_2_SO_4_ 0.01%, and incubated O/N at −20 °C. The following day, Doxo concentration was measured by spectrofluorimetry and compared with a predetermined calibration curve.

### Cells

The human monocytic THP-1 cell line was obtained from Istituto Zooprofilattico (Brescia, Italy). Cell culture media and all supplements were purchased from Sigma. For pyrogen test experiments, THP-1 cells were diluted to 10^6^ cells/mL in RPMI 1640 containing 2 mM l-glutamine, 0.1 mg/mL streptomycin, 100 IU/mL penicillin, and 50 μM 2-mercaptoethanol, supplemented with 10% heated-inactivated fetal calf serum and cultured at 37 °C in a 5% CO_2_ incubator.

For cytokine storm, blood samples were taken by venous puncture with sodium citrate 0.5 M as the anticoagulant. Healthy subjects (*n* = 5) were selected according to the guidelines of the Italian Health authorities and to the Declaration of Helsinki principles and signed an informed consent (average 40 y, min 25 max 53). Criteria for exclusion were the use of medication known to affect the immune system, i.e., steroids, or patients suffering from malignancies, inflammations, and infections. Blood samples were diluted 1:10 in cell culture medium RPMI 1640 (Sigma, St Louis, USA) containing 2 mM l-glutamine, 0.1 mg/mL streptomycin, and 100 IU/mL penicillin, cultured at 37 °C in a 5% CO_2_ incubator and freshly incubated with testing agents.

### Cytokine Production

Cells were treated with HFn, HFnX, HCC, and HCX at a predetermined concentration of 500 μg/mL. Free Doxo, D-HFn, and D-HCX were incubated at an equivalent Doxo concentration of 3 μg/mL. This concentration was selected as it is the average drug concentration found in blood during routine clinical use of Doxo. The positive control PHA (5 μg/mL) was used. Cytokine release was studied after incubating fresh primary blood cells for 24 and 48 h with all testing agents. Cytokine production was assessed in cell-free supernatants by specific commercially available sandwich ELISA (R&D System for TNF-α; ImmunoTools for IL-6 and IFN-γ). Cell-free supernatants obtained by centrifugation at 2500 rpm for 5 min were stored at −20 °C until measurement. Results are expressed as pg/mL, calculated by interpolating absorbance readings with a calibration curve.

A pyrogen test was performed by incubating THP-1 cells with ferritin-based testing agents for 3 and 24 h. TNF-α levels were evaluated as described above. To investigate the possible presence of endotoxin in stimulating TNF-α, the testing agents were preincubated with polymyxin B sulfate (15 μg/mL final concentration) for 1 h at 37 °C and then added to THP-1 cells. LPS 0.1 μg/mL was used as the positive control.

### Treatments with Doxo-Loaded Nanodrugs and Cell Viability

Cell viability was assessed by flow cytometric evaluation of propidium iodide (PI)-stained cells following 24 h of treatment with Doxo, D-HFnX, and D-HCX at different concentrations. After incubation, the cells were centrifuged at 1500 rpm for 5 min and suspended in 0.5 mL of PBS containing 1 μg/mL PI. The percentage of positive cells was analyzed using a NovoCyte 3000 flow cytometer, and data were quantified using NovoFlow software. Results are expressed as % of viable cells.

### Statistical Analysis

All experiments using THP-1 cells were performed at least three times, with representative results shown. Five donors were used for the whole blood assay. Statistical analysis was performed using GraphPad InStat version 5.0a for Macintosh (GraphPad Software, San Diego, CA, USA). For multiple comparisons, ANOVA was performed with the Dunnett test. For blood samples, one-way ANOVA and paired Student’s *t*-test were used. Differences were considered significant at *p* ≤ 0.05.

## Supporting Information Available

Detailed description of HFn, HFnX, HCC, and HCX production methods; detailed description of the optimization process followed for the HCC induction step; and viability assessment and pyrogen test of free and nanodrug-loaded Doxo (PDF)

## Notes

Availability of data and material: data are available in a publicly accessible repository after publication https://doi.org/10.13130/RD_UNIMI/LJT2YN.

## Abbreviations

NP(s): nanoparticle(s)
LPS: lipopolysaccharides
TLR4: Toll-like receptor 4
HFn: H-ferritin
TfR1: transferrin receptor 1
HFnX: endotoxin-free HFn
EU: endotoxin unit
mL: milliliters
mg: milligrams
SDS-PAGE: sodium dodecyl sulfate-polyacrylamide gel electrophoresis
TEM: transmission electron microscopy
mM: millimolar
h: hours
min: minutes
°C: Celsius degree
v/v: volume to volume ratio
L: liters
Nm: nanometers
μg: micrograms
PHA: phytohemagglutinin
IFN-γ: interferon gamma
IL-6: interleukin 6
TNF-α: tumor necrosis factor alpha
pg: picograms
poly: polymyxin B
HCC: H-ferritin produced in ClearColi
HCX: endotoxin-free H-ferritin produced in ClearColi
O/N: overnight
IPTG: isopropyl β-d-1-thiogalactopyranoside
HT: heat-treated
F1-6: fractions 1–6
LAL: limulus amebocyte lysate
Doxo: doxorubicin
D-HFnX: doxorubicin-loaded HFnX nanodrugs
D-HCX: doxorubicin-loaded HCX nanodrugs
Rec: recovery
mol no.: number of molecules
