# Supplementary material for: In Vitro Immunoreactivity Evaluation of H-Ferritin-Based Nanodrugs
Source: Bioconjug Chem. 2023 Feb 24;34(5):845–55. doi: 10.1021/acs.bioconjchem.3c00038 (PMC10197070; doi:10.1021/acs.bioconjchem.3c00038)
Supplement: Supplementary file 1 — bc3c00038_si_001.pdf [file bc3c00038_si_001.pdf]

SUPPORTING INFORMATION

In vitro immunoreactivity evaluation of H-Ferritin based nanodrugs

Leopoldo Sitia[#†], Valentina Galbiati[#‡], Arianna Bonizzi[†], Marta Sevieri[†], Marta Truffi[§], Mattia Pinori[†], Emanuela Corsini[‡], Marina Marinovich[‡], Fabio Corsi[§†], and Serena Mazzucchelli[*†].

†: Department of Biomedical and Clinical Sciences, Università degli studi di Milano, 20157, Milan, Italy

‡: Department of Pharmacological and Biomolecular Sciences, Università degli studi di Milano, 20133, Milan, Italy

§: Istituti Clinici Scientifici Maugeri IRCCS, 27100 Pavia, Italy

#: These authors contributed equally to this work.

*: Correspondence to: serena.mazzucchelli@unimi.it, +390250319689, Department of Biomedical and Clinical Sciences, Università degli studi di Milano, via G.B. Grassi 74, 20157, Milan, Italy.

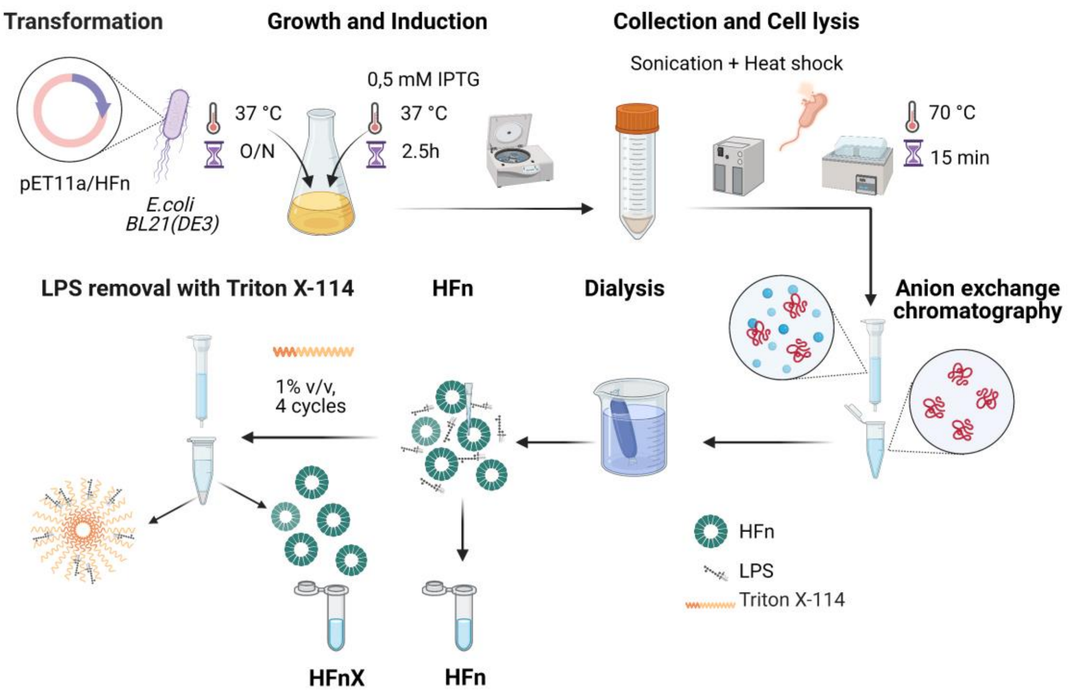

Figure S1: Detailed HFn and HFnX production method.

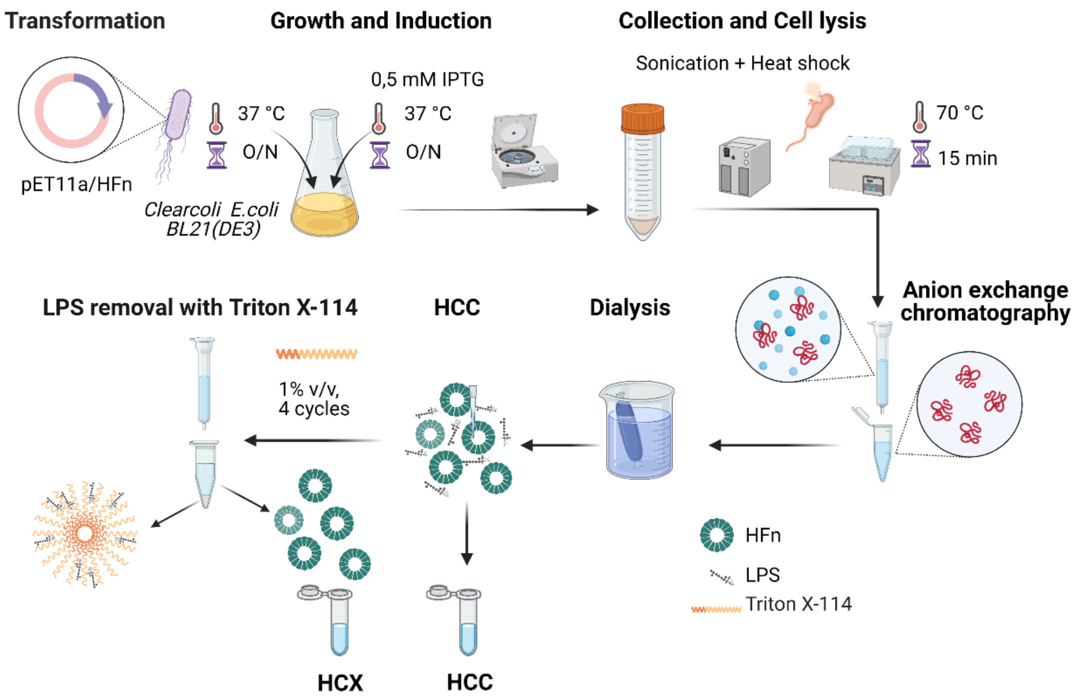

Figure S2: Detailed HCC and HCX production method.

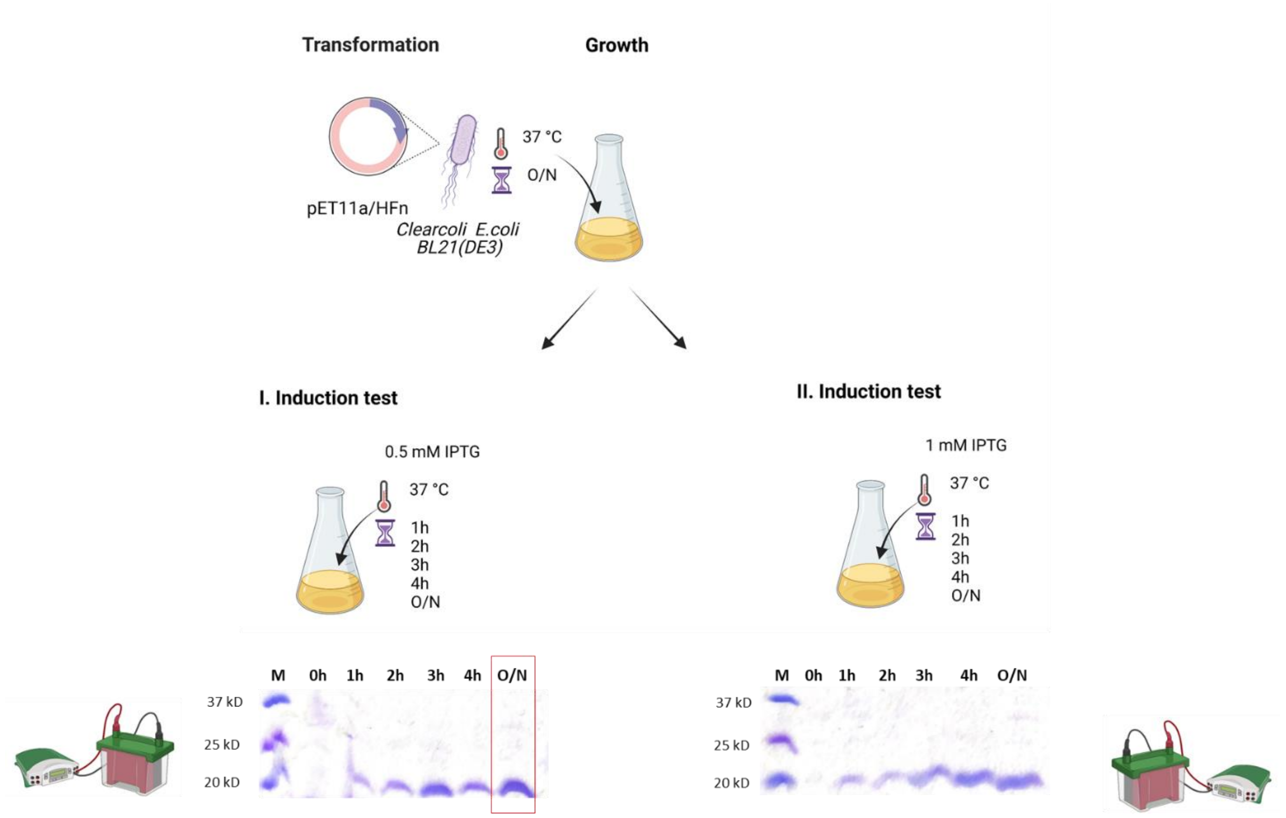

Figure S3: Induction Test. To evaluate the expression of HCC we carried out induction tests with two different concentrations of IPTG (0.5 mM and 1 mM) and at different times (1h, 2h,3h,4h and overnight). The results obtained show a greater HCC expression using 0.5mM IPTG overnight.

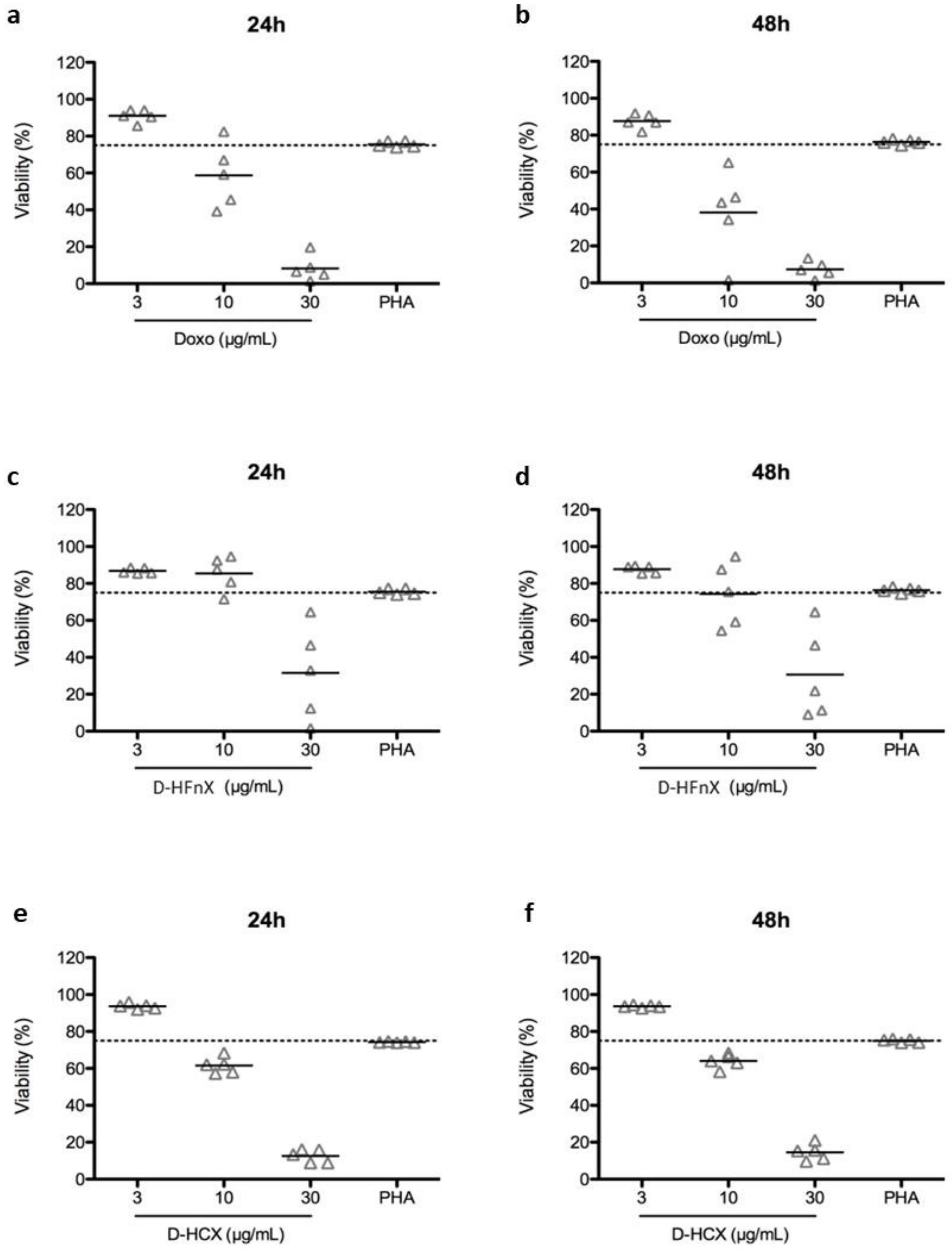

Figure S4 – Viability assessment of dose-response experiments with doxorubicin loaded nanodrugs. Whole blood was diluted 1:10 in culture media and exposed to doxorubicin (Doxo) (**a, b**), doxorubicin-HFnX (D-HFnX) (**c, d**) and doxorubicin-HCX (D-HCX) (**e, f**) at three different concentrations (3, 10 and 30 µg/mL) for 24 and 48 h to assess viability. PHA (5 µg/mL) was also compared as it was used as positive control in cytokine storm experiments. Each dot represents independent donors (*n=5*). The dot line represents the 75% of cell viability (CV75).

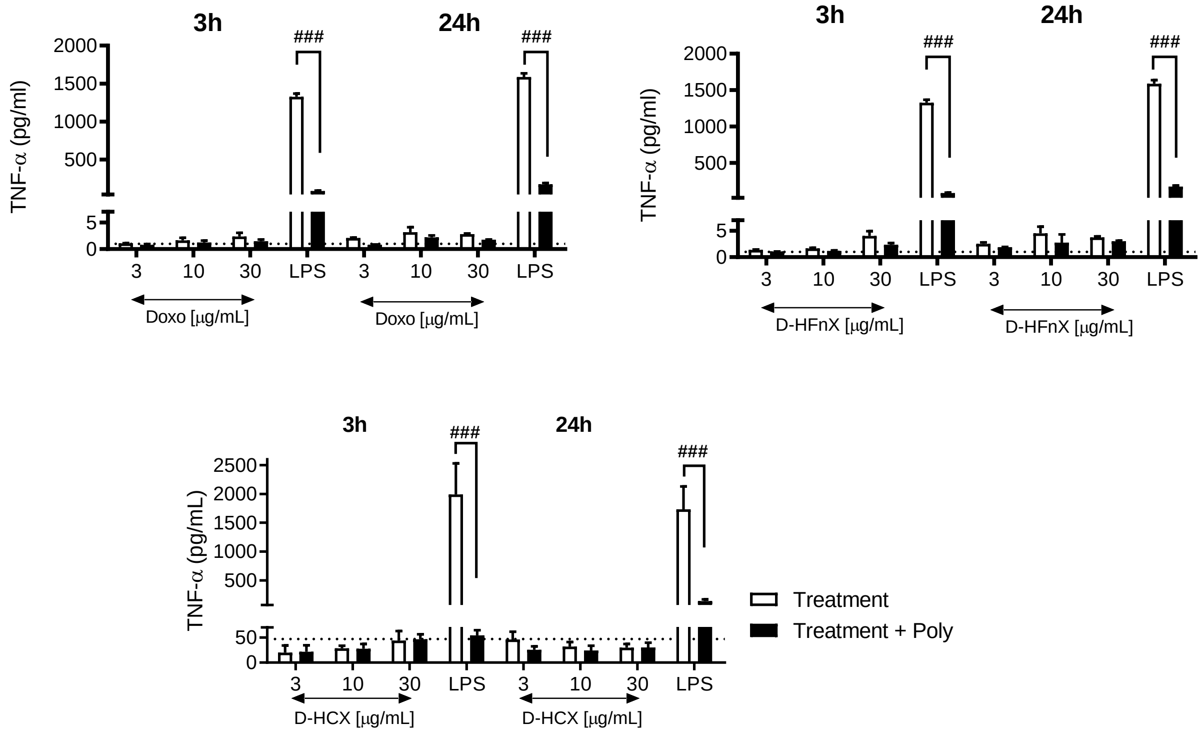

Figure S5 – Pyrogen test of Doxo and Doxo loaded nanodrugs on THP-1 cells. The THP-1 cells were exposed to increasing concentrations of free doxorubicin (Doxo), D-HFnX, D-HCX, and LPS (0.1 μg/mL as positive control) for 3 and 24h to assess TNF-α release (pg/mL). Each column represents three independent experiments (*n=3*). Statistical analysis was performed by Dunnett's Multiple comparison test, with $^{\#}p < 0.05$ and $^{\#\#\#}p < 0.001$ *versus* respective exposed group.
